# Autophagy Is Involved in Stellate Ganglion Block Reversing Posthemorrhagic Shock Mesenteric Lymph-Mediated Vascular Hyporeactivity

**DOI:** 10.3389/fphys.2021.728191

**Published:** 2021-09-21

**Authors:** Chen Wang, Hui-Bo Du, Zhen-Ao Zhao, Jia-Yi Zhai, Li-Min Zhang, Chun-Yu Niu, Zi-Gang Zhao

**Affiliations:** ^1^Institute of Microcirculation, Hebei North University, Zhangjiakou, China; ^2^Pathophysiology Experimental Teaching Center of Basic Medical College, Hebei North University, Zhangjiakou, China; ^3^Key Laboratory of Critical Disease Mechanism and Intervention in Hebei, Hebei Medical University & Hebei North University, Shijiazhuang & Zhangjiakou, China; ^4^Basic Medical College, Hebei Medical University, Shijiazhuang, China

**Keywords:** hemorrhagic shock, vascular reactivity, stellate ganglion block, mesenteric lymph, vascular smooth muscle cells, autophagy

## Abstract

**Objective:** The aim of this study was to clarify the role of autophagy in stellate ganglion block (SGB) reversing posthemorrhagic shock mesenteric lymph (PHSML)-mediated vascular hyporeactivity.

**Methods:** Hemorrhagic shock model in conscious rats was employed to observe the effects of SGB (0.2 ml of 0.25% ropivacaine hydrochloride hydrate) and autophagy inhibitor 3-methyladenine (3-MA; 30 mg/kg) on the vascular reactivity of second-order rat mesenteric arteries *in vitro*, while the effects of PHSML (1 ml/kg) and autophagy agonist rapamycin (Rapa, 10 mg/kg) on the beneficial effect of SGB were investigated. The cellular viability, contractility, and autophagy-related protein expressions in vascular smooth muscle cells (VSMCs) were detected following treatments of PHSML, PHSML obtained from the rats that underwent hemorrhagic shock plus SGB (PHSML-SGB), and PHSML plus 3-MA (5 mM), respectively.

**Results:** Hemorrhagic shock significantly decreased the vascular reactivity to gradient norepinephrine (NE), which is reversed by the SGB treatment and 3-MA administration. On the contrary, PHSML intravenous infusion and Rapa administration inhibited the vascular contractile responses in rats that underwent hemorrhagic shock plus SGB treatment. PHSML treatment significantly inhibited the cellular viability and contractility in VSMCs, increased the expressions of LC3-II and Beclin 1, and decreased the expression of p62, along with opposite appearances in these indices following PHSML-SGB treatment. In addition, 3-MA counteracted the adverse roles of PHSML in these indices in VSMCs.

**Conclusion:** SGB inhibits PHSML-mediated vascular hyporeactivity by reducing the excessive autophagy in VSMCs.

## Introduction

Hemorrhagic shock is a common and critical emergency in intensive care medicine, which is caused by acute massive bleeding following multiple traumas, peptic ulcers, obstetrical accidents, surgical accidents, and other factors (Cannon, 2018). Since bleeding could not be duly controlled and patients do not receive active and effective fluid resuscitation, the patients with massive hemorrhage will lead to shock and multiple organ injury characterized by high mortality. Previous studies have shown that the death of about 1.8 million critically ill patients annually worldwide is due to hemorrhagic shock (Cannon, 2018). Vascular hyporeactivity becomes a key reason during hemorrhagic shock leading to intractable hypotension and refractory shock (Duan et al., 2015). Therefore, it is necessary to strengthen the research on the pathogenesis of severe hemorrhagic shock-induced vascular hyporeactivity and to explore new therapeutic measures.

Many studies demonstrated that posthemorrhagic shock mesenteric lymph (PHSML) return is a key link and basic pathway of hemorrhagic shock-induced uncontrolled inflammatory response and multiple organ injury (Deitch, 2012; Rocha-e-Silva, 2016; Nunns et al., 2018). Previous studies found that the blockage of PHSML by mesenteric lymphatic ligation or mesenteric lymph drainage improves the vascular reactivity in rats with hemorrhagic shock, and PHSML reduces the vascular reactivity to norepinephrine (NE) *in vitro* of vascular rings isolated from normal rats (Zhao et al., 2012). However, mesenteric lymphatic ligation or mesenteric lymph drainage is difficult to apply in clinical practice. Therefore, it is of positive significance to find an alternative way for the prevention and treatment of severe hemorrhagic shock. The sympathetic nervous system and parasympathetic nervous system play important roles in the regulation of heart rate, rhythm, and responsiveness (Witt Chance et al., 2017). Since stellate ganglion (SG) is functionally the peripheral sympathetic ganglia, our previous studies found that stellate ganglion block (SGB) significantly prolongs the survival time and improves the intestinal barrier function in rats following hemorrhagic shock. However, whether SGB treatment improves vascular hyporeactivity remains unclear.

Autophagy, as a process of cell renewal, cells use “self-feeding” to recover cytoplasmic contents, is a unique membrane transport process, which plays an important role to maintain cell homeostasis and survival (Choi et al., 2013). However, the excessive autophagy activated by a strong stimulus usually causes “autophagic” cell death (Shiomi et al., 2014). The previous studies have shown that hemorrhagic shock induced excessive autophagy in hepatocytes (Shi et al., 2021), as well as hepatic ischemia/reperfusion injury (Go et al., 2015). Vascular smooth muscle cells (VSMCs) participate in the physiological and pathological changes, such as vascular contraction. However, the following issues are unknown: whether severe hemorrhagic shock induces excessive autophagy in VSMCs, what is the relationship between excessive autophagy of VSMCs and vascular hyporeactivity, whether PHSML return-mediated vascular hyporeactivity is related to excessive autophagy of VSMCs, and whether SGB that improves vascular reactivity is related to the inhibition of the autophagy of VSMCs.

Hence, we hypothesized that SGB reversed the PHSML-mediated vascular hyporeactivity through the inhibition of autophagy. To clarify this hypothesis, we established the hemorrhagic shock model in conscious rats and investigated the effects of SGB, PHSML infusion, autophagy inhibitors, and agonists on the vascular reactivity of isolated mesenteric arterioles. Furthermore, we used the VSMCs and detected the effects of PHSML, PHSML obtained from the rats that underwent hemorrhagic shock plus SGB treatment (PHSML-SGB), autophagy inhibitors, and agonists on the cellular viability, contractility, and autophagy-related protein expression. As a result, this study will provide new experimental data for the prevention and treatment of vascular hyporeactivity after hemorrhagic shock and will expand the clinical application of SGB.

## Materials and Methods

### Experimental Animal and Procedure

Fifty-two healthy adult male rats, weighing 280–320 g, were purchased from Sibefu Biotechnology Co., Ltd., Beijing, China, and were raised in the animal room (temperature 21–25°C, relative humidity 40–60%, light rhythm alternates day and night). The animal experiment was approved by the Animal Ethics Committee of the Hebei North University (No.2018-1-9-10). Before the experiment, the animals were fasted for 8 h and drank freely.

**Figure 1:**
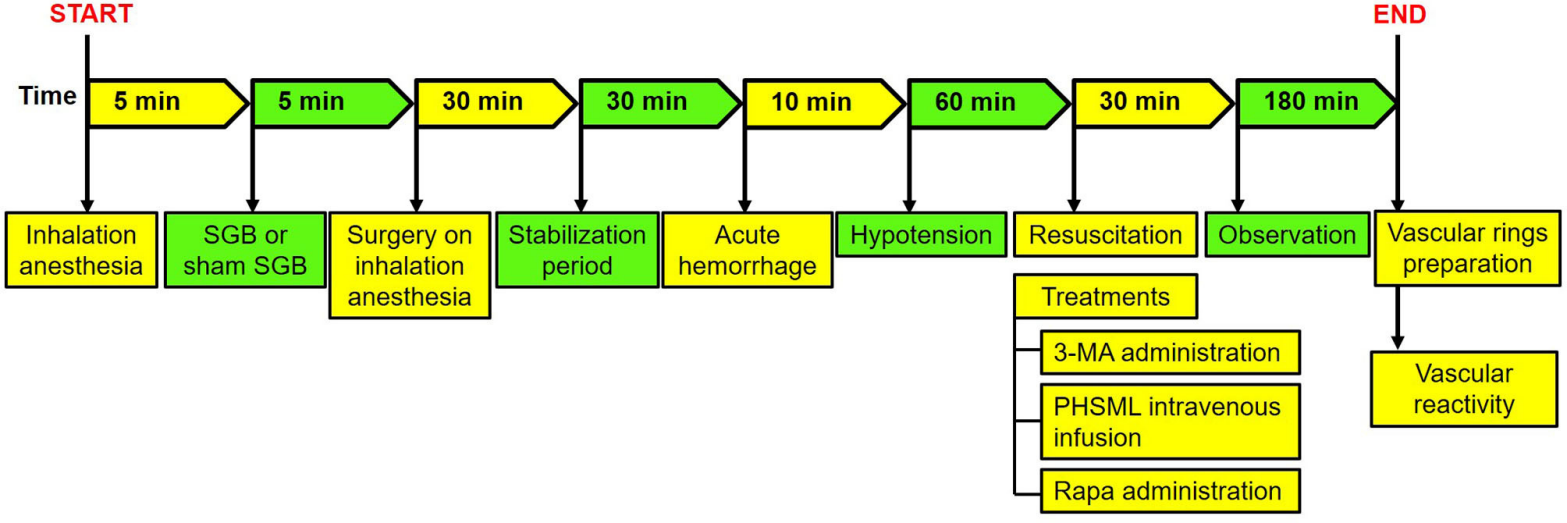
The surgical procedure of hemorrhagic shock model in conscious rats. SGB, stellate ganglion block; PHSML, posthemorrhagic shock mesenteric lymph.

The animal experiment was performed as follows: Part I: 10 rats were randomly divided into the Shock and Shock + SGB groups (*n* = 5/group). These rats were used to establish the controlled hemorrhagic shock model for the collection of PHSML and PHSML-SGB, respectively. Also, a lymph sample was used for subsequent animal experiments and cytology experiments. Part II: The rats were randomly divided into seven groups, namely, Sham, Sham+SGB, Shock, Shock + SGB, Shock plus 3-methyladenine (3-MA) intraperitoneal injection (30 mg/kg) (Zhang et al., 2015) (Shock + 3-MA), Shock + SGB plus rapamycin (Rapa) intraperitoneal injection (10 mg/kg) (James et al., 2016) (Shock + SGB + Rapa), and Shock + SGB plus intravenous infusion of PHSML (1 ml/kg) (Yin et al., 2020) (Shock + SGB + PHSML), 6 rats in each group. The experimental procedures are shown in Table 1. The hemorrhagic shock model was established in conscious rats (Figure 1) for the investigation of the mechanism by which SGB reversing vascular hyporeactivity caused by PHSML-mediated autophagy. The detailed procedure of the hemorrhagic shock model in conscious rats was shown in the “Establishment of hemorrhagic shock model in conscious rats” section.

**Table 1:** Experimental procedure in various groups in animal experiment.

**Group**	**SGB**	**Hemorrhage**	**Resuscitation**	**Administration of 3-MA**	**Administration of Rapa**	**Intravenous injection of PHSML**
Sham	-	-	-	-	-	-
Sham + SGB	+	-	-	-	-	-
Shock	-	+	+	-	-	-
Shock + SGB	+	+	+	-	-	-
Shock + 3-MA	-	+	+	+	-	-
Shock + SGB + Rapa	+	+	+	-	+	-
Shock + SGB + PHSML	+	+	+	-	-	+

*SGB, stellate ganglion block (0.2 ml of 0.25% ropivacaine hydrochloride hydrate); 3-MA, 3-methyladenine (autophagy inhibitor, 30 mg/kg); Rapa, rapamycin (autophagy activator, 10 mg/kg); PHSML, posthemorrhagic shock mesenteric lymph*.

### Implement of SGB

After recording the body weight of rats, anesthesia was induced by the inhalation of 3% VOL isoflurane using a small animal anesthetic machine (MATRX VMR, Midmark Corporation, Dayton, Ohio, USA). Under inhalation anesthesia, the right SGB was performed according to the previous reports (Abdi and Yang, 2005; Wang et al., 2017; Zhang et al., 2019; Yin et al., 2020). In brief, 0.2 ml of 0.25% ropivacaine hydrochloride hydrate was injected by a posterior percutaneous approach. After isoflurane inhalation withdraw for about 5 min, then the rats naturally awakened, the right droopy eyelid was used as a positive sign of SGB successfully. To Sham SGB, the same procedures were performed except for injecting the same amount of saline.

### Collection of PHSML

After SGB treatment or not, rats were anesthetized with pentobarbital sodium for the establishment of a hemorrhagic shock model and the collection of mesenteric lymph according to our previous study (Zhao et al., 2012; Wang et al., 2020). In brief, rats received an acute hemorrhage (40 ± 2 mmHg of mean artery pressure for 60 min) and resuscitation with shed whole blood and Ringer's solution at a 1:1 ratio. Then, polyethylene tubing was aseptically cannulated to the mesenteric lymphatic duct for mesenteric lymph drainage for 3 h. After centrifugation for removing cells, the mesenteric lymph sample was stored in a refrigerator at −80°C and used for the next experiments.

### Establishment of Hemorrhagic Shock Model in Conscious Rats

After SGB or Sham SGB, rats received inhalational anesthesia again and were fixed on the operating table of small animals in the supine position and connected with the oxygen mask to maintain anesthesia. Then, surgical procedures (Figure 1) were performed under inhalation anesthesia for the establishment of hemorrhagic shock according to the previous study (Yin et al., 2020) as the following.

After preoperative hair removal and skin disinfectant, a skin incision of about 0.5 cm was cut down at the neck, and then the process of femoral cutdown was performed on both sides to separate both femoral arteries and left femoral vein from the surrounding tissue. Three PE-50 soft catheters about 100 cm long were conducted into the femoral arteries and femoral vein and were traveled subcutaneously from the femoral surgical incision to the neck incision to create a subcutaneously perforating tube channel (to prevent rats destroy the soft catheters under wakefulness). Subsequently, the 1% heparin (10 mg/kg, 1 ml/kg) was injected through the left femoral vein for systemic anticoagulation, and then 0.1% heparin sodium solution was injected into the three catheters for anticoagulation and maintaining unobstructed. Finally, all surgical incisions were sutured and disinfected, penicillin (1 mg/kg) was injected intramuscularly to prevent infection, and lidocaine injection was performed to relieve pain. When the inhalation anesthesia was stopped after the operation, the rats could regain consciousness within 5 min. The time of operation was controlled within 30 min.

After being awakened, the rats were put into a round transparent bucket. The catheter conducted into the femoral arteries were attached to the PowerLab biological signal acquisition system (ADInstruments, Bella Vista NSW, Australia) for monitoring mean arterial blood pressure (MAP) in real-time and a syringe that was fixed onto a program-controlled withdrawal-infusion machine (NE-1000, New Era Pump Systems Inc., Farmingdale, NY, USA) for bloodletting, respectively. The femoral vein catheter was attached to the other program-controlled withdrawal-infusion machine for resuscitation.

After a 30 min-stabilization period in the bucket, blood was withdrawn at a rate of 0.6 ml/min. The MAP was reduced to 40 mmHg in the first 10 min. In the next 1 h, the MAP was maintained at 40 ± 2 mmHg through bloodletting and blood transfusion. During hypotension, the shed blood was stored in a 20-ml-syringe connected automatic withdrawal-infusion machine, at room temperature. After hypotension of 1 h, the whole blood and equal Ringer's solution were mixed and infused into the femoral vein at a constant speed. At the same time, the administrations of 3-MA (30 mg/kg) and Rapa (10 mg/kg) and the intravenous injection of PHSML were performed according to the experimental procedures. The time of fluid resuscitation was controlled within 30 min. At 3 h after fluid resuscitation, the inhalational anesthesia was carried out for subsequent experiments.

### Analyses of Vascular Reactivity

The rat mesentery was collected at the corresponding time points in each group, and the second-order rat mesenteric arteries with a diameter of 80–120 μm and a distance of 8–10 cm from the cecum were selected. Four vascular rings of 2 mm long were prepared, and each ring was threaded into two steel wires with a length of 2 cm and a diameter of 0.0396 mm in a bath contains 5 ml pretreated physiological saline solution ([PSS] 119 nM NaCl, 4.7 mM KCl, 1.17 mM MgSO_4_, 25 mM NaHCO_3_, 1.2 mM KH_2_PO_4_, 0.03 mM ethylenediaminetetraacetic acid (EDTA), 5.5 mM dextrose, 2.5 mM CaCl_2_, pH 7.3–7.4) at 4°C, which continuously filled with a mixed gas of 5% CO_2_ and 95% O_2_. The vascular ring was stabilized for 5 min leading to a natural tension-free state. Then, the bath was then slowly heated to 37°C. The standardized procedures for zeroing and activation were performed with NE or with the stimulation of high potassium solution, respectively, according to the previous reports (Mulvany and Halpern, 1977; Zhao et al., 2014). After 10 min of activation, it was washed again with preheated PSS to make the tension curve return to baseline. After stabilizing for 5 min, NE with gradient concentration was added to the bath and the final concentration of NE in the bath was increased gradually to 0.1, 0.3, 1, 3, 10, 30, and 100 μM. The vascular constriction response of the artery rings to each concentration of NE was recorded using PowerLab System through a force transducer (Duan et al., 2020). The difference in tension between maximal value and baseline following stimulation with each concentration of NE was used as the value of vascular reactivity at this concentration. Vascular reactivity data were recorded from at least two arterial rings per animal. The maximal contraction (Emax) of the vascular ring under all concentrations of NE was obtained, and pD2 (–log [50% effective concentration, EC50] of NE) was obtained from concentration-response curves. The Emax and pD2 were used for the further evaluation of vascular reactivity.

### Analysis of Cell Proliferation

The VSMCs of the rat isolated from mesentery (RAT-CELL-0089, PriCells, Wuhan, China) were cultured in the DMEM-F12 medium (C11330500BT, Gibco, Grand Island, NY, USA) supplemented with 20% fetal bovine serum (04-001-1ACS, Gibco), penicillin (100 units/ml), and streptomycin (100 units/ml). Cells between the fourth to sixth passages were maintained in the presence of 5% CO_2_ at 37°C in a humidified atmosphere. To verify the effect and mechanism of SGB on PHSML-mediated cell proliferation dysfunction, VSMCs were treated with PHSML (4%), PHSML-SGB (4%), PHSML plus 3-MA (5 mM) (Peng et al., 2017), and PHSML-SGB plus Rapa (100 nM) (Chen et al., 2018), respectively. Dimethysulfoxide (DMSO) was used as a control. Cell Counting Kit-8 (CCK-8, E1008, Applygen Technologies Inc., Beijing, China) was used to measure cell viability according to the protocol of the manufacturer. In brief, cells at the exponential stage were seeded into 96-well plates at a density of 5,000 cells/well in a final volume of 100 μl and were cultured for 48 h. After cells adhering to the wall, the culture medium was changed to various lymph and/or drug for co-incubation for 6 h. Then, 10 μl of CCK-8 solution was added to each well for a 4-h incubation. Cell viability was calculated by measuring the absorbance at 450 nm. All experiments were repeated three times, which means three independent experiments were used for cellular proliferation analysis.

### Analysis of Cellular Contractile Response

The VSMCs at the exponential stage were seeded into the apical chamber of Transwell (3415, Corning, Beijing, China) (3.0 μm of pore size and a 0.33 cm^2^ coated with Matrigel) at a density of 1 × 10^5^/ml in a final volume of 200 μl, while 600 μl of 20% DMEM-F12 complete medium was added in the basolateral chamber. After co-incubation for 72 h, the cells were exposed to various treatments for 6 h according to the experimental design. After treatments, in each well plate, 5 μl of fluorescein-labeled bovine serum albumin (4 mg/ml) and 100 μl of NE solution (5 mM) were added to the apical chamber, respectively. Then, 100 μl of culture medium in the basolateral chamber was harvested at 5, 10, 15, 25, 35, 45, and 60 min, respectively. After collecting 100 μl of medium from the basolateral chamber, 100 μl of 20% DMEM-F12 complete medium was added to it. The fluorescence intensity of the culture medium was measured by the fluorescent module of SpectraMax M3 (Molecular Devices, San Francisco, CA, USA) at an excitation spectrum of 495 nm and an absorption spectrum of 520 nm. The cellular contractile response was evaluated by the cumulative filtration to fluorescein-labeled bovine serum albumin at per time and 60 min, which was calculated according to the accumulative filtration of fluorescein to 5 μl fluorescein-labeled bovine serum albumin: Cumulative filtration to fluorescein-labeled albumin = Cumulative fluorescein value/Total fluorescein value. It should be pointed out that the single concentration of NE was used for the analysis of cellular contractile response, i.e., no gradient concentration in isolated vascular reactivity, which is accorded to the relevant literature (Essler et al., 1998; Li et al., 2008; Yang et al., 2010).

### Analyses of Immunofluorescence

Cells at the exponential stage were seeded into the confocal dish at a density of 1 × 10^5^/ml in a final volume of 200 μl and cultured for 18–24 h. After the cells adhered to the wall, the culture medium was discarded, drugs were added, and the cells were cultured for 6 h. Then, cells were washed with phosphate-buffered saline (PBS) and fixed with 4% paraformaldehyde for 30 min. After being permeabilized with Triton X-100, cells were blocked with 5% normal bovine serum albumin for 30 min and incubated with the primary monoclonal anti-LC3-II antibody (ab192890, Abcam, Cambridge, UK) overnight at a dilution of 1:500. Then, the dishes were washed to remove the primary antibody and rinsed with PBS, and those were incubated with a fluorescein isothiocyanate secondary antibody for 90 min. The dishes were mounted after being stained with 4',6-diamidino-2-phenylindole (DAPI) for 5 min and analyzed under a laser confocal microscope (FV1200, Olympus, Tokyo, Japan).

### Analyses of Western Blotting

The VSMCs were assigned into the control group (vehicle), PHSML group, PHSML-SGB group, and PHSML+3-MA group. Cells at the exponential stage were seeded into 6-well plates at a density of 1 × 10^5^/ml in a final volume of 200 μl and cultured for 24–48 h. After the confluence of the cells reached 80%, the culture medium was discarded, and the cells were treated with various treatments for 6 h. Then, the cells were washed with PBS three times and lysed in 100 μl RIPA. After a brief sonication and centrifugation, the supernatants were collected for protein concentration measurement by the BCA kit (P1511, Applygen Technologies Inc., Beijing, China). The proteins in each group were separated using sodium dodecyl sulfate-polyacrylamide gel electrophoresis (SDS-PAGE) and transferred to the polyvinylidene fluoride (PVDF) membrane. Transferring membrane was performed with a constant current of 250 mA for 35 min for LC3-II and 300 mA for 80 min for p62, Beclin 1, and β-actin. After being blocked with 5% milk, the membranes were incubated with primary antibodies, such as anti-LC3-II (ab192890, Abcam, Cambridge, UK, 1:1,000), anti-p62 (5114S, Cell Signaling Technology, Danvers, MA, USA, 1:1,000), anti-Beclin 1 (ab207612, Abcam, Cambridge, UK, 1:1,000), and anti-β-actin (E2317, Santa CruZ Biotechnology, Delaware, CA, USA, 1:5,000). After overnight at 4°C, the membranes were washed and incubated with secondary antibodies (1:2,000) at room temperature for 1 h. Enhanced chemiluminescence (ECL) was used for signal development. ImageQuant LAS 4000 (GE Co., Boston, MA, USA) was used to capture the chemiluminescence. The analysis was conducted using Quantity One software (version 4.6.2, Bio-Rad Co., Hercules, CA, USA), and the relative protein levels were expressed as the intensity ratios of the target protein to β-actin.

### Statistical Analyses

All experiments were independently performed at least three times. The data were expressed as mean ± SEM. The statistical analysis was performed using SPSS version 22 (IBM, Armonk, NY, USA). Differences among groups were analyzed using the one-way ANOVA and the *post hoc* test with the Student-Newman-Keul (SNK) test. In addition, two-way repeated measures ANOVA was used to evaluate the overall trend of vascular reactivity. *P* < 0.05 was considered to be statistically significant.

## Results

### SGB Treatment Improves Vascular Reactivity of Second-Order Rat Mesenteric Arteries in Rats With Hemorrhagic Shock

**Figure 2:**
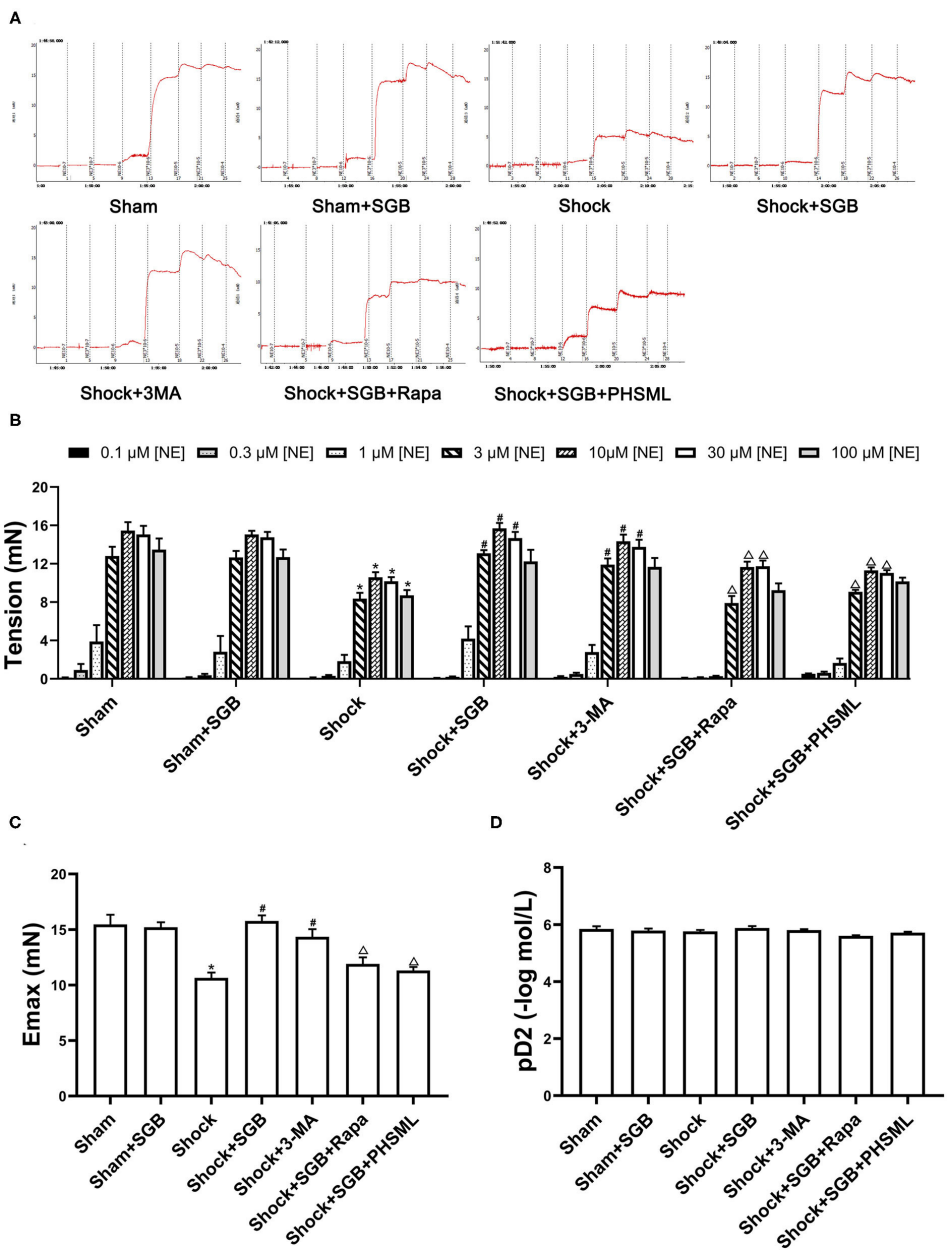
Effect of stellate ganglion block (SGB) treatment, autophagy inhibitor or activator administrations, and posthemorrhagic shock mesenteric lymph (PHSML) intravenous infusion on the mesenteric microvascular contractile reactivity to norepinephrine (NE) following hemorrhagic shock. SGB was performed with 0.2 ml of 0.25% ropivacaine hydrochloride hydrate; 3-methyladenine (3-MA, autophagy inhibitor) and rapamycin (Rapa, autophagy activator) administrations were performed with doses of 30 and 10 mg/kg, respectively. **(A)** Original tracings of vascular reactivity of mesenteric micro-artery in rats. **(B)** The changes of vascular reactivity following NE stimulation, the final concentrations of NE in the bath were 0.1, 0.3, 1, 3, 10, 30, and 100 μM, respectively. **(C)** Results of Emax (maximal contractive tension). **(D)** Results of pD2 (negative logarithm (–log) of NE concentration produced 50% maximal contractive effect). The data are expressed as the mean ± SEM (*n* = 6). Differences among groups were analyzed using the one-way ANOVA and the *post hoc* test with the Student-Newman-Keul (SNK) test. The overall trend of vascular reactivity was evaluated using two-way repeated measures ANOVA. *P < 0.05 compared with the Sham group; ^#^*P* < 0.05 compared with the Shock group; ^Δ^*P* < 0.05 compared with the Shock + SGB group.

First, the results (Figures 2A,B) showed that there was no significant difference in the reactivity to each concentration of NE between the Sham and Sham + SGB groups (*P* > 0.05). The reactivity to NE (3, 10, 30, and 100 μM) in the Shock group was significantly lower than that in the Sham group (*P* < 0.05). The reactivity to NE (3, 10, and 30 μM) in the Shock + SGB group was significantly higher than that in the Shock group (*P* < 0.05), and there was no significant difference between the Shock + SGB and Sham groups (*P* > 0.05). Second, the results showed that the vascular reactivity to NE (3, 10, and 30 μM) in the Shock + 3-MA group was significantly enhanced compared with the Shock group (*P* < 0.05). The reactivity of mesenteric arterioles to NE (3, 10, and 30 μM) in the Shock + SGB+Rapa group was significantly lower than that in the Shock + SGB group (*P* < 0.05). In addition, this index in the Shock + SGB + PHSML group was significantly lower than that in the Shock + SGB group (NE concentration of 3, 10, and 30 μM, *P* < 0.05).

The repeated measures ANOVA showed that there was no significant difference in the contractile response to NE between the Sham group and the Sham + SGB group as a whole (*P* > 0.05), along with no significant difference between the Shock + SGB group and the Sham group as a whole (*P* > 0.05). The hemorrhagic shock significantly decreased the contractile response than that in the Sham group (*P* < 0.05), which was reversed by SGB treatment and 3-MA administration (*P* < 0.05). The effect of SGB treatment on the contractile response was abrogated by Rapa administration and intravenous infusion of PHSML (*P* < 0.05). In addition, the results of Emax in vascular contraction showed a similar change (Figure 2C) to the vascular reactivity as described earlier. However, there was no statistical difference in pD2 among these groups (Figure 2D).

### Effect of SGB on PHSML Inhibiting the Cell Variability

**Figure 3:**
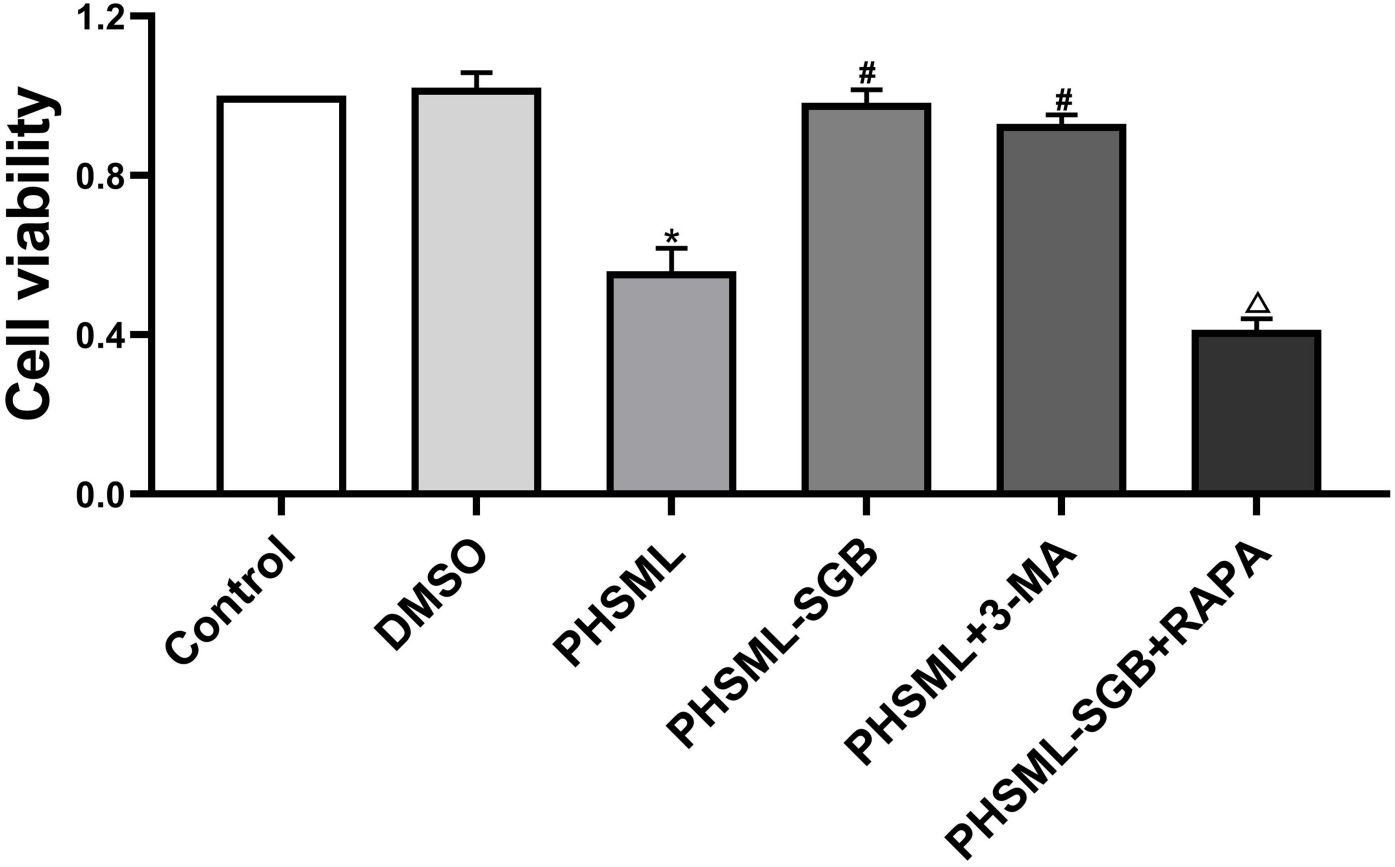
Effect of SGB and autophagy drugs on the viability of vascular smooth muscle cells (VSMCs) injured by PHSML. The cells were treated with PHSML (4%), PHSML-SGB (PHSML obtained from the rats underwent hemorrhagic shock plus SGB treatment, 4%), PHSML plus 3-MA (autophagy inhibitor, 5 mM), and PHSML-SGB plus Rapa (autophagy activator, 100 nM) for 6 h, respectively. The cell viability was detected by the Cell Counting Kit-8 (CCK-8) method. The data are presented as mean ± SEM (*n* = 6). Differences among groups were analyzed using the one-way ANOVA and the *post hoc* test with the SNK test. *P < 0.05 compared with the control group; ^#^*P* < 0.05 compared with the PHSML group; ^Δ^*P* < 0.05 compared with the PHSML-SGB group.

As shown in Figure 3, PHSML treatment significantly inhibited the cell activity of VSMCs compared with the control group (*P* < 0.05), which was reversed by the 3-MA administration. At the same time, the cell variability after PHSML-SGB treatment was significantly increased than that in the PHSML group (*P* < 0.05). At the same time, mTOR inhibitor Rapa inhibited the cell variability of VSMCs in the PHSML-SGB group (*P* < 0.05).

### Effect of SGB on PHSML Inhibiting the Cellular Contractility

**Figure 4:**
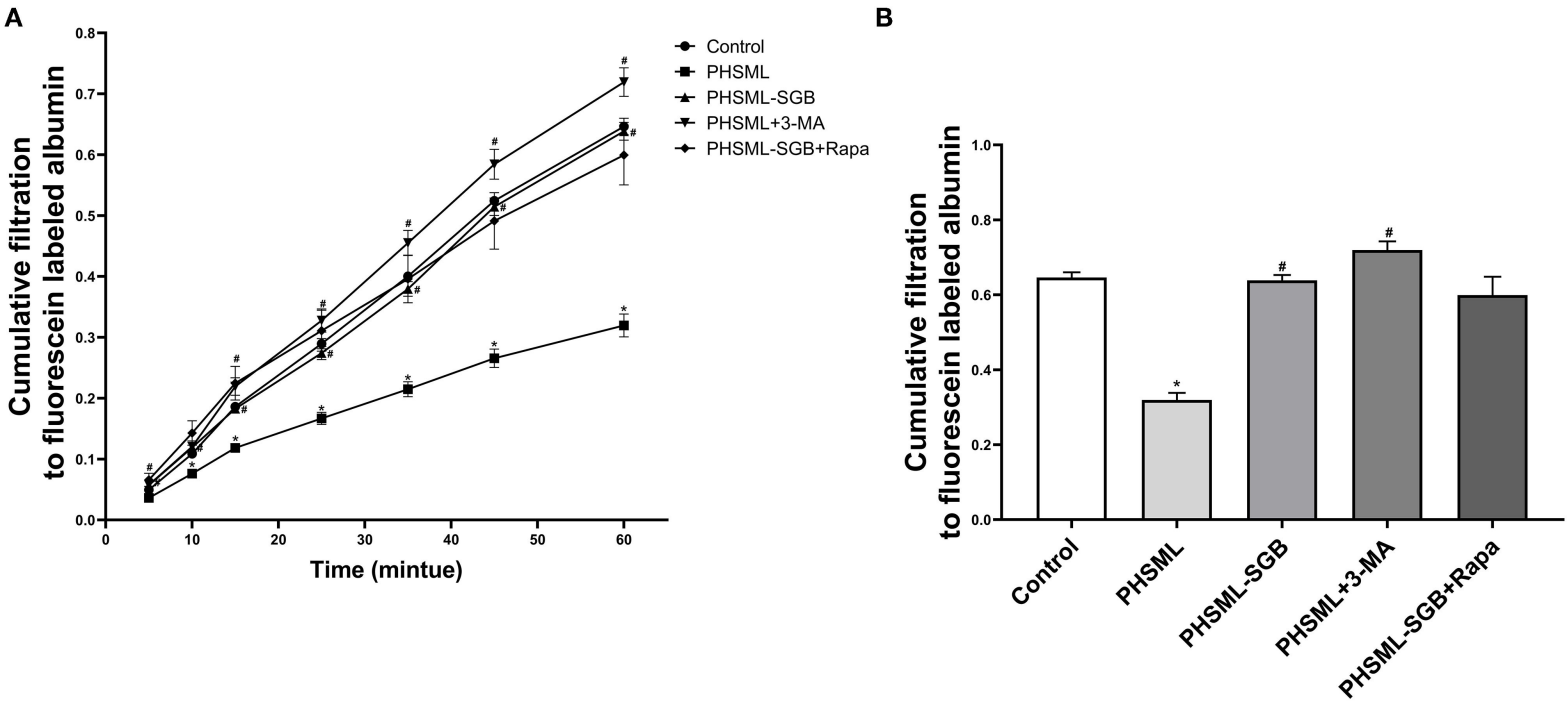
Effect of SGB and autophagy drugs on the contractility of VSMCs injured by PHSML. The cells were treated with PHSML, PHSML-SGB (PHSML obtained from the rats underwent hemorrhagic shock plus SGB treatment), PHSML plus 3-MA (autophagy inhibitor, 5 mM), and PHSML-SGB plus Rapa (autophagy activator, 100 nM) for 6 h, respectively. The cellular contractility to noradrenaline (final concentration of 0.5 mM) was detected using the Transwell chamber and expressed with the cumulative filtration to fluorescence-labeled albumin. **(A)** Time-cumulative filtration curves. **(B)** Cumulative filtration to fluorescence-labeled albumin at 60 min. The data are presented as mean ± SEM (*n* = 6). Differences among groups were analyzed using the one-way ANOVA and the *post hoc* test with the SNK test. *P < 0.05, compared with the control group; ^#^*P* < 0.05 compared with the PHSML group.

As shown in Figure 4, the cumulative transmittances of VSMCs to fluorescence-labeled albumin at 10, 15, 25, 35, 45, and 60 min in the PHSML group were significantly lower than that in the control group (*P* < 0.05), which was significantly increased in the PHSML + 3-MA group at 5, 15, 25, 35, 45, and 60 min (*P* < 0.05). In addition, this index at all time points in the PHSML-SGB group was significantly higher than that in the PHSML group (*P* < 0.05). However, there was no significant difference in the cumulative transmittance of fluorescence-labeled albumin between PHSML-SGB+Rapa and PHSML-SGB groups (*P* > 0.05).

### Effect of SGB on PHSML Activating Autophagy in VSMCs

**Figure 5:**
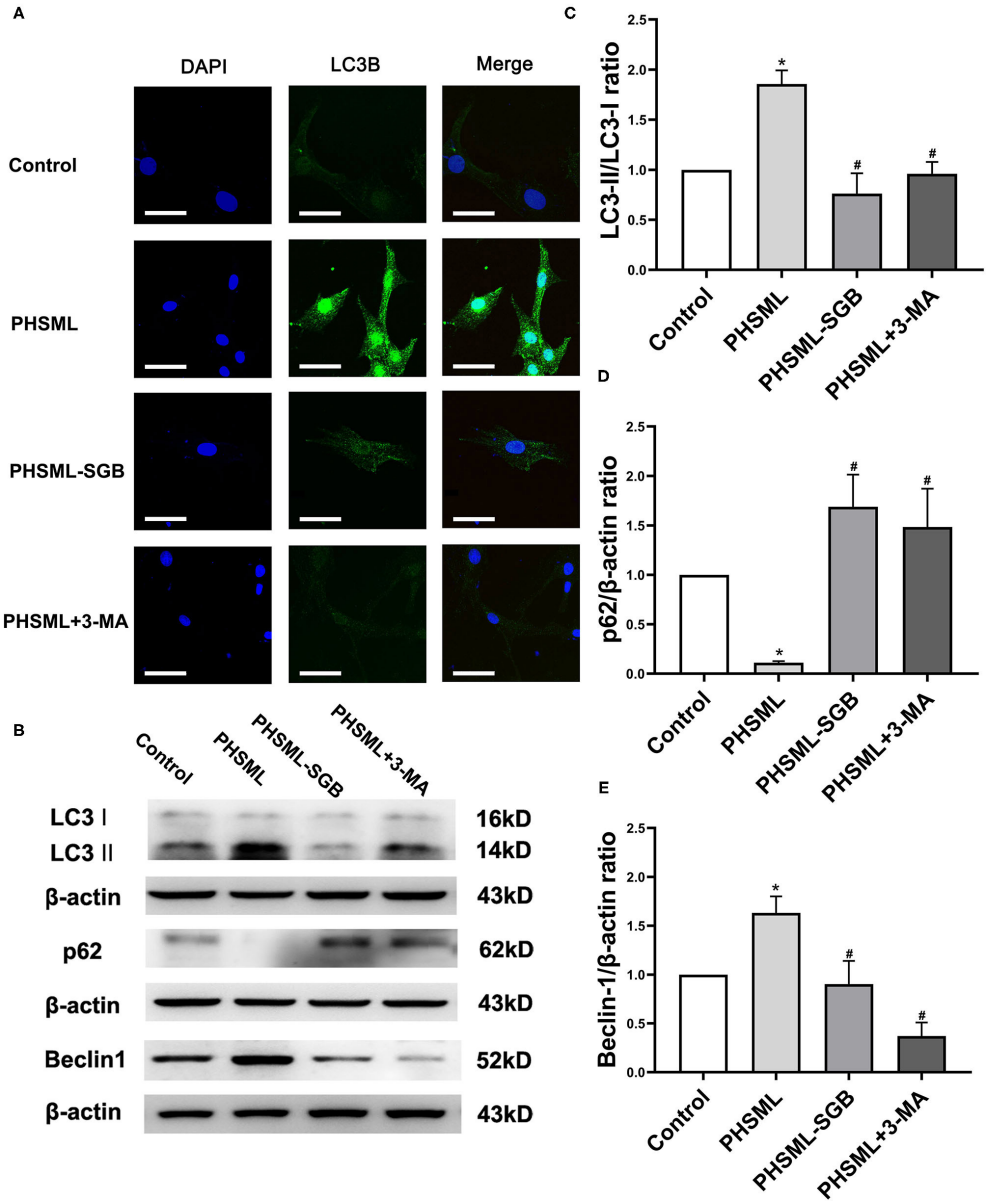
Effects of SGB on PHSML-induced expressions of autophagy proteins in VSMCs. The cells were treated with PHSML, PHSML-SGB (PHSML obtained from the rats underwent hemorrhagic shock plus SGB treatment), and PHSML plus 3-MA (autophagy inhibitor, 5 mM) for 6 h, respectively. **(A)** The representativeness images of the localization and expression of LC3B protein in VSMCs under an Olympus laser confocal microscope. Bar = 50 μm. **(B)** The representativeness images of the expressions of marker proteins of autophagy in VSMCs using the Western blotting method. **(C–E)** The expressions of LC3-II, p62, and Beclin 1 in VSMCs. The densitometric values were normalized to β-actin. The data are expressed as the mean ± SEM (*n* = 3). Differences among groups were analyzed using the one-way ANOVA and the *post hoc* test with the SNK test. *P < 0.05 compared with the control group; #*P* < 0.05 compared with the PHSML group.

Immunofluorescence was used to detect the expression of LC3-II in VSMCs after different treatments. The representative images showed that the green fluorescence was greatly increased in cells treated with PHSML compared with the control group, while the green fluorescence in PHSML-SGB and PHSML + 3-MA groups was significantly decreased compared with the PHSML group, respectively (Figure 5A). The results of Western blotting (Figures 5B,C) showed that the expression of LC3-II and Beclin 1 in the PHSML group was significantly higher than that in the control group, while the expression of p62 was significantly decreased (*P* < 0.05). In contrast, PHSML-SGB and 3-MA treatment decreased the LC3-II and Beclin 1 expression induced by PHSML (*P* < 0.05) and increased the expression of p62 (*P* < 0.05).

## Discussion

Studies have shown that vascular hyporeactivity is an important factor in intractable hypotension after hemorrhagic shock (Duan et al., 2015), and autophagy affects the structure and function of VSMCs. Previous studies in our laboratory have shown that SGB can significantly prolong the survival time of rats with hemorrhagic shock (Zhang et al., 2019), and the PHSML return mediates vascular hyporeactivity (Zhao et al., 2012). Based on the regulation of autophagy homeostasis, this study investigated the intestinal lymphatic mechanism by which SGB treatment in improving vascular reactivity in hemorrhagic shock. Our data demonstrated that SGB inhibited PHSML-mediated vascular hyporeactivity by reducing excessive autophagy of VSMCs.

To reduce the effects of anesthesia on animal metabolism and to make the experimental process closer to the clinic, this article used a hemorrhagic shock model in conscious rats (Singh et al., 1991) to observe the changes in vascular reactivity. The application of inhalation anesthesia not only ensures the ethical norms of experimental animals and makes the animals undergo surgery at the minimum injury degree but also helps the animals to wake up naturally and reduces the effects of anesthesia on all aspects of physiological functions of animals to the maximum extent. Compared with the traditional hemorrhagic shock in rats under anesthesia, the animals lose blood quickly in the awake state, which is almost similar to the actual situation of hemorrhagic shock patients and better simulates the physiological and pathophysiological changes during hemorrhagic shock.

Initially, based on the mature implementation of SGB, the effect of SGB on vascular hyporeactivity in rats with hemorrhagic shock was observed by using the four-channel vascular-tension-measuring instrument *in vitro*. The results showed that the vascular contractile response to gradient NE was significantly decreased in rats with hemorrhagic shock, while SGB treatment significantly improved the vascular reactivity at increasing concentrations of NE. The results confirmed the favorable effect of SGB on improving vascular reactivity in rats with hemorrhagic shock, which may be beneficial to tissue blood perfusion. Therefore, whether SGB can improve the blood perfusion of organs (e.g., intestines and kidneys) needs investigation in the future.

The hemorrhagic shock-induced vascular hyporeactivity is associated with acidosis, uncontrolled inflammatory response, oxidative stress, and other factors caused by continuous ischemia and hypoxia (Duan et al., 2015). These factors further cause the adrenergic receptor desensitization, membrane hyperpolarization, and calcium desensitization of VSMCs (Zhao et al., 2002; Zhao, 2005; Duan et al., 2015). A previous study found that PHSML return was involved in the process of vascular hyporeactivity caused by hemorrhagic shock (Zhao et al., 2012). To investigate whether the effect of SGB on improving vascular reactivity in hemorrhagic shock is related to PHSML, this study performed the intravenous infusion of PHSML into rats with hemorrhagic shock which were pretreated with SGB. The results showed that the intravenous infusion of PHSML reduced the effect of SGB on the contractile response of rats with hemorrhagic shock, suggesting that the hemorrhagic shock-induced vascular hyporeactivity was at least partially mediated by PHSML return. It is well-known that endothelial cells and smooth muscle cells interact to form blood vessels, and VSMCs are the only cell type in the middle layer of blood vessels (Lacolley et al., 2012). VSMCs participate in the contraction and diastole of blood vessels through the interaction of Ca^2+^-calmodulin and then play an important role in blood pressure regulation, oxygen, and nutrient transport (Webb, 2003). Therefore, based on the results of the animal model, PHSML was further applied to VSMCs cultured *in vitro*. The results showed that PHSML treatment significantly inhibited the cellular activity and contraction ability to NE, confirming the effect of PHSML on reducing vascular reactivity at the cellular level. More importantly, after PHSML-SGB treatment, the cellular activity and the contraction ability were significantly stronger than that in the PHSML group, which confirmed that the effect of SGB on vascular hyporeactivity was realized through the intestinal lymphatic pathway. However, whether SGB causes the change of PHSML components, thus improving vascular reactivity, needs further study.

Autophagy connects human physiological and pathological processes. On the one hand, autophagy can clear senile cells and tumor cells and can play a positive protective role. On the other hand, excessive autophagy is an important factor leading to cell death, tissue necrosis, and organ damage (Todde et al., 2009). Studies have shown that excessive autophagy is involved in the structural damage of hepatocytes after hepatic ischemia-reperfusion injury (Shen et al., 2013), as well as in the pathogenesis of blood loss, posttraumatic brain injury, and organ injury after sepsis (Chen et al., 2012; Ho et al., 2016; Ratliff et al., 2016). These studies suggest that excessive autophagy is one of the mechanisms in histiocyte injury. Studies have shown that under certain conditions, the activation of autophagy may cause VSMC necrosis and apoptosis (Krueger et al., 2003), suggesting that autophagy plays an important role in vascular diseases by regulating VSMC function. To further explore the role of autophagy homeostasis in the improvement of vascular reactivity after hemorrhagic shock by SGB, autophagy-related drugs were used in rats with hemorrhagic shock *in vivo* and VSMCs *in vitro*. The results showed that 3-MA, a specific inhibitor of autophagy, significantly improved the vasoconstrictive reactivity of isolated arterioles of rats with hemorrhagic shock to gradient NE, and the autophagy agonist Rapa reduced the beneficial effect of SGB on improving the vasoconstriction of rats with hemorrhagic shock. Furthermore, 3-MA treatment significantly inhibited the decrease of cell viability and contractile response caused by PHSML. Rapa significantly inhibited the cell viability of VSMCs treated with PHSML-SGB. These results suggested that the effect of SGB on improving the vascular reactivity following a hemorrhagic shock is related to the inhibition of autophagy.

In fact, LC3-II is a biomarker of autophagosome formation. The content of LC3-II in cells can reflect the degree of autophagy, and Beclin 1 can regulate the formation and maturation of autophagosomes by binding to a variety of proteins (Corona Velazquez and Jackson, 2018). The p62 protein, which is also known as sequestosome 1, is an autophagy receptor (Lamark et al., 2017). The p62 protein directly binds to LC3 and GABARAP family proteins through specific sequence motifs and participates in autophagy. Due to the accumulation of p62 during autophagy inhibition and the decrease of p62 protein level during the activation of autophagy, p62 is often used as a marker protein to study autophagy flux (Bjørkøy et al., 2009). To clarify the mechanism that SGB enhances the activity and contractile response of VSMCs treated with PHSML by inhibiting autophagy, the expression of autophagy-specific biomarker LC3-II in VSMCs was detected by immunofluorescence, and the ratio of autophagy marker protein LC3 II/LC3 I (i.e., LC3 II/I), Beclin 1, and p62 expression was detected. The results show that PHSML treatment significantly increased the expressions of LC3-II and Beclin 1 and decreased the p62 expression, which suggested that PHSML activated the autophagy of VSMCs. In contrast, PHSML-SGB treatment decreased the LC3-II and Beclin 1 expression induced by PHSML and increased the expression of p62, which suggested that SGB treatment inhibits the activation of the autophagy of VSMCs by PHSML. More importantly, 3-MA treatment also inhibited the effect of PHSML in the autophagy-expressed protein of VSMCs. To sum up, the inhibitory effect of SGB on PHSML-mediated vascular hyporeactivity is achieved by inhibiting the autophagy of VSMCs.

It should be pointed out that vascular contractility following hemorrhagic shock was studied by various methods, such as pressor response to NE *in vivo* (Zhao et al., 2012; Li et al., 2014), a reduced percentage of vascular diameter after NE administration *in vivo* (Li et al., 2014), and isolated vascular contractile response to gradient concentrations of NE *in vitro* (Duan et al., 2020; Yue et al., 2021). While all these methods clarified that hemorrhagic shock induced vascular hyporeactivity in various ways. In general, after acute hemorrhage and fluid resuscitation, NE administration *in vivo* will induce lung edema to a certain degree; therefore, this study investigated the vascular reactivity with isolated vascular rings *in vitro* but no pressor response and vascular diameter response *in vivo*. However, the universality of SGB improving vascular contractility following hemorrhagic shock needs further investigation using the model without resuscitation. Meanwhile, various vasoactive substances affect vascular contraction and relaxation, e.g., NE, epinephrine, high K^+^, thromboxane, endothelin-1, serotonin, acetylcholine, and nitric oxide. This study only used NE as a tool drug for the observation of vascular constriction; therefore, more vasoactive substances should be used to observe the changes in vasoconstriction in future experiments. Furthermore, these results presented an inconsistent phenomenon between vascular reactivity and VSMC contraction. In brief, Rapa injection inhibited the beneficial effect of SGB on the vascular reactivity of isolated arteriole from rats with hemorrhagic shock, but Rapa administration failed to inhibit the cellular constriction of VSMCs treated with PHSML-SGB *in vitro*. The main reasons might be related to different environments of animal and cellular experiments. It would be better to seek a more direct method of observing the contractility of VSMCs in future experiments.

## Conclusion

**Figure 6:**
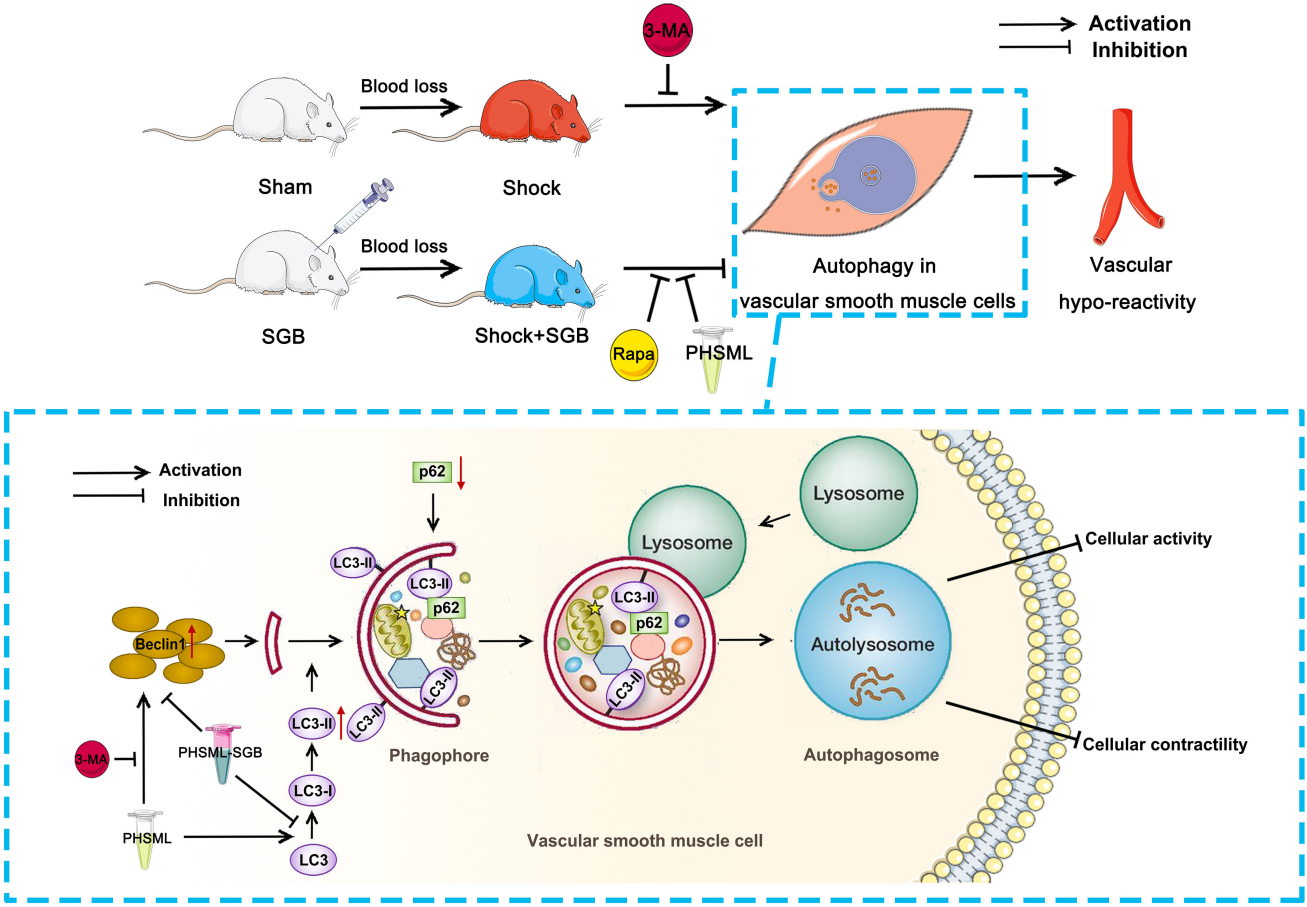
The SGB inhibits the PHSML-mediated vascular hyporeactivity by reducing the excessive autophagy in VSMCs. SGB treatment and autophagy inhibitor 3-MA administration abolish hemorrhagic shock-induced vascular hyporeactivity, while the intravenous infusion of PHSML reverses the beneficial effects of SGB. Furthermore, PHSML incubation inhibits cellular contractile response and induces autophagy of VSMCs with evidence of increased expressions of LC3-II/LC3-I and Beclin 1 and decreased expression of p62, which was reversed by 3-MA treatment. Importantly, PHSML obtained from the rats underwent hemorrhagic shock plus SGB treatment (PHSML-SGB) has a reverse effect compared with PHSML.

These results suggest that SGB improved the vascular reactivity of rats with hemorrhagic shock, which is related to the intestinal lymphatic pathway and its mechanism is related to the inhibition of excessive autophagy in VSMCs (Figure 6). The results of this study not only enriched the mechanism of vascular reactivity after hemorrhagic shock by autophagy but also provided the experimental basis for the clinical application of SGB in the prevention and treatment of severe shock. It should be pointed out that the occurrence of vascular hyporeactivity in hemorrhagic shock is related to the factors such as desensitization or decreased activity of the VSMC adrenergic receptors, cell membrane hyperpolarization, and cellular calcium desensitization (Duan et al., 2015). In this study, we only studied the effect of SGB on vascular reactivity after hemorrhagic shock from the perspective of the autophagy of VSMC. However, whether SGB can improve the expression of the VSMC adrenergic receptors, can reverse the hyperpolarization of the VSMC cell membrane, and can increase the calcium sensitivity of VSMCs remains to be further observed.

## Data Availability Statement

The original contributions presented in the study are included in the article/Supplementary Material, further inquiries can be directed to the corresponding authors.

## Ethics Statement

The animal study was reviewed and approved by Animal ethics committee of Hebei North University.

## Author Contributions

CW, H-BD, Z-AZ, J-YZ, and L-MZ performed majority of the animal experiment and laboratory work. CW acquired and analyzed the data. Z-GZ and C-YN were involved in the conception and design of the study, data interpretation, and critically revised the manuscript. All authors contributed to the article and approved the submitted version.

## Funding

This study was supported by the Natural Science Foundation of Hebei Province to Z-GZ (H2020405012) and the National Natural Science Foundation of China to C-YN (No. 81770492).

## Conflict of Interest

The authors declare that the research was conducted in the absence of any commercial or financial relationships that could be construed as a potential conflict of interest.

## Publisher's Note

All claims expressed in this article are solely those of the authors and do not necessarily represent those of their affiliated organizations, or those of the publisher, the editors and the reviewers. Any product that may be evaluated in this article, or claim that may be made by its manufacturer, is not guaranteed or endorsed by the publisher.

## References

[B1] Abdi S. Yang Z. (2005). A novel technique for experimental stellate ganglion block in rats. Anesth. Analg. 101, 561–565. 10.1213/01.ANE.0000159169.12425.50 16037176

[B2] Bjørkøy G. Lamark T. Pankiv S. Øvervatn A. Brech A. Johansen T. (2009). Monitoring autophagic degradation of p62/SQSTM1. Methods Enzymol. 452, 181–197. 10.1016/S0076-6879(08)03612-4 19200883

[B3] Cannon J. W. (2018). Hemorrhagic shock. N. Engl J. Med. 378, 370–379. 10.1056/NEJMra1705649 29365303

[B4] Chen C. W. Chen T. Y. Tsai K. L. Lin C. L. Yokoyama K. K. Lee W. S.. (2012). Inhibition of autophagy as a therapeutic strategy of iron-induced brain injury after hemorrhage. Autophagy 8, 1510–1520. 10.4161/auto.21289 22909970

[B5] Chen Y. Zhang H. Liu H. Li K. Jia M. Su X. (2018). High glucose upregulated vascular smooth muscle endothelin subtype B receptors via inhibition of autophagy in rat superior mesenteric arteries. Ann. Vasc. Surg. 52, 207–215. 10.1016/j.avsg.2018.02.028 29758325

[B6] Choi A. M. Ryter S. W. Levine B. (2013). Autophagy in human health and disease. N. Engl. J. Med. 368, 651–662. 10.1056/NEJMra1205406 23406030

[B7] Corona Velazquez A. F. Jackson W. T. (2018). So many roads: the multifaceted regulation of autophagy induction. Mol. Cell Biol. 38, e00303–e00318. 10.1128/MCB.00303-18 30126896 PMC6189458

[B8] Deitch E. A. (2012). Gut-origin sepsis: evolution of a concept. Surgeon 10, 350–356. 10.1016/j.surge.2012.03.003 22534256 PMC3413774

[B9] Duan C. Wang L. Zhang J. Xiang X. Wu Y. Zhang Z.. (2020). Mdivi-1 attenuates oxidative stress and exerts vascular protection in ischemic/hypoxic injury by a mechanism independent of Drp1 GTPase activity. Redox Biol. 37:101706. 10.1016/j.redox.2020.101706 32911435 PMC7490562

[B10] Duan C. Yang G. Li T. Liu L. (2015). Advances in vascular hyporeactivity after shock: the mechanisms and managements. Shock 44, 524–534. 10.1097/SHK.0000000000000457 26263436

[B11] Essler M. Amano M. Kruse H. J. Kaibuchi K. Weber P. C. Aepfelbacher M. (1998). Thrombin inactivates myosin light chain phosphatase via Rho and its target Rho kinase in human endothelial cells. J. Biol. Chem. 273, 21867–21874. 10.1074/jbc.273.34.21867 9705325

[B12] Go K. L. Lee S. Zendejas I. Behrns K. E. Kim J. S. (2015). Mitochondrial dysfunction and autophagy in hepatic ischemia/reperfusion injury. Biomed. Res. Int., 2015:183469. 10.1155/2015/183469 26770970 PMC4684839

[B13] Ho J. Yu J. Wong S. H. Zhang L. Liu X. Wong W. T.. (2016). Autophagy in sepsis: degradation into exhaustion? Autophagy 12, 1073–1082. 10.1080/15548627.2016.1179410 27172163 PMC4990998

[B14] James M. H. Quinn R. K. Ong L. K. Levi E. M. Smith D. W. Dickson P. W.. (2016). Rapamycin reduces motivated responding for cocaine and alters GluA1 expression in the ventral but not dorsal striatum. Eur. J. Pharmacol. 784, 147–154. 10.1016/j.ejphar.2016.05.013 27181066

[B15] Krueger K. D. Hunter W. J. 3rd DelCore M. G. Agrawal D. K. (2003). Calphostin C as a rapid and strong inducer of apoptosis in human coronary artery smooth muscle cells. Int. Immunopharmacol. 3, 1751–1759. 10.1016/S1567-5769(03)00206-6 14636826

[B16] Lacolley P. Regnault V. Nicoletti A. Li Z. Michel J. B. (2012). The vascular smooth muscle cell in arterial pathology: a cell that can take on multiple roles. Cardiovasc. Res. 95, 194–204. 10.1093/cvr/cvs135 22467316

[B17] Lamark T. Svenning S. Johansen T. (2017). Regulation of selective autophagy: the p62/SQSTM1 paradigm. Essays Biochem. 61, 609–624. 10.1042/EBC20170035 29233872

[B18] Li T. Liu L. Liu J. Ming J. Xu J. Yang G.. (2008). Mechanisms of Rho kinase regulation of vascular reactivity following hemorrhagic shock in rats. Shock 29, 65–70. 10.1097/shk.0b013e318063e477 17666953

[B19] Li T. Xiao X. Zhang J. Zhu Y. Hu Y. Zang J.. (2014). Age and sex differences in vascular responsiveness in healthy and trauma patients: contribution of estrogen receptor-mediated Rho kinase and PKC pathways. Am. J. Physiol. Heart Circ. Physiol. 306, H1105–1115. 10.1152/ajpheart.00645.2013 24531808

[B20] Mulvany M. J. Halpern W. (1977). Contractile properties of small arterial resistance vessels in spontaneously hypertensive and normotensive rats. Circ. Res. 41, 19–26. 10.1161/01.RES.41.1.19 862138

[B21] Nunns G. R. Stringham J. R. Gamboni F. Moore E. E. Fragoso M. Stettler G. R.. (2018). Trauma and hemorrhagic shock activate molecular association of 5-lipoxygenase and 5-lipoxygenase-Activating protein in lung tissue. J. Surg. Res. 229:262–270. 10.1016/j.jss.2018.03.023 29936999 PMC6020847

[B22] Peng Y. Q. Xiong D. Lin X. Cui R. R. Xu F. Zhong J. Y.. (2017). oestrogen inhibits arterial calcification by promoting autophagy. Sci. Rep. 7:3549. 10.1038/s41598-017-03801-x 28615727 PMC5471178

[B23] Ratliff E. P. Barekat A. Lipinski M. M. Finley K. D. (2016). Brain trauma and autophagy: what flies and mice can teach us about conserved responses. Autophagy 12, 2256–2257. 10.1080/15548627.2016.1221565 27560096 PMC5103346

[B24] Rocha-e-Silva M. (2016). Cardiovascular effects of shock and trauma in experimental models. A review. Braz. J. Cardiovasc. Surg. 31, 45–51. 10.5935/1678-9741.20150065 27074274 PMC5062691

[B25] Shen M. Lu J. Dai W. Wang F. Xu L. Chen K.. (2013). Ethyl pyruvate ameliorates hepatic ischemia-reperfusion injury by inhibiting intrinsic pathway of apoptosis and autophagy. Mediators Inflamm. 2013:461536. 10.1155/2013/461536 24453420 PMC3886226

[B26] Shi Y. Greven J. Guo W. Luo P. Xu D. Wang W.. (2021). Trauma-hemorrhage stimulates immune defense, mitochondrial dysfunction, autophagy, and apoptosis in pig liver at 72 h. Shock 55, 630–639. 10.1097/SHK.0000000000001556 32826806

[B27] Shiomi M. Miyamae M. Takemura G. Kaneda K. Inamura Y. Onishi A.. (2014). Induction of autophagy restores the loss of sevoflurane cardiac preconditioning seen with prolonged ischemic insult. Eur. J. Pharmacol. 724, 58–66. 10.1016/j.ejphar.2013.12.027 24374197

[B28] Singh G. Chaudry K. I. Chudler L. C. O'Neill P. J. Chaudry I. H. (1991). Measurement of D-xylose gut absorptive capacity in conscious rats. Am. J. Physiol. 261(5 Pt 2), R1313–1320. 10.1152/ajpregu.1991.261.5.R1313 1951781

[B29] Todde V. Veenhuis M. van der Klei I. J. (2009). Autophagy: principles and significance in health and disease. Biochim. Biophys. Acta 1792, 3–13. 10.1016/j.bbadis.2008.10.016 19022377

[B30] Wang Q. Chi Z. F. Wei D. Zhao Z. A. Zhang H. Zhang L. M.. (2020). Transcriptome analysis revealed inflammation is involved in the impairment of human umbilical vein endothelial cells induced by post-hemorrhagic shock mesenteric lymph. Front. Immunol. 11:1717. 10.3389/fimmu.2020.01717 33013823 PMC7509150

[B31] Wang W. Shi W. Qian H. Deng X. Wang T. Li W. (2017). Stellate ganglion block attenuates chronic stress induced depression in rats. PLoS ONE 12:e0183995. 10.1371/journal.pone.0183995 28859148 PMC5578491

[B32] Webb R. C. (2003). Smooth muscle contraction and relaxation. Adv. Physiol. Educ. 27, 201–206. 10.1152/advances.2003.27.4.201 14627618

[B33] Witt Chance M. Bolona L. Kinney Michelle O. Moir C. Ackerman Michael J. Kapa S. uraj Asirvatham Samuel J.. (2017). Denervation of the extrinsic cardiac sympathetic nervous system as a treatment modality for arrhythmia. EP Europace 19, 1075–1083. 10.1093/europace/eux011 28340164

[B34] Yang G. Xu J. Li T. Ming J. Chen W. Liu L. (2010). Role of V1a receptor in AVP-induced restoration of vascular hyporeactivity and its relationship to MLCP-MLC20 phosphorylation pathway. J. Surg. Res. 161, 312–320. 10.1016/j.jss.2009.01.005 19577256

[B35] Yin M. Li Z. H. Wang C. Li Y. Zhang H. Du H. B.. (2020). Stellate ganglion blockade repairs intestinal mucosal barrier through suppression of endoplasmic reticulum stress following hemorrhagic shock. Int. J. Med. Sci., 17, 2147–2154. 10.7150/ijms.47662 32922175 PMC7484657

[B36] Yue Y. X. Zhai J. Y. Du H. B. Jiang L. N. Zhang L. M. Wang C.. (2021). Estrogen enhances the microvascular reactivity through rhoa-rock pathway in female mice during hemorrhagic shock. Shock. 10.1097/SHK.0000000000001776 33756501

[B37] Zhang J. Lin X. R. Zhang Y. P. Zhang L. M. Du H. B. Jiang L. N.. (2019). Blockade of stellate ganglion remediates hemorrhagic shock-induced intestinal barrier dysfunction. J. Surg. Res. 244, 69–76. 10.1016/j.jss.2019.06.007 31279996

[B38] Zhang Y. L. Zhang J. Cui L. Y. Yang S. (2015). Autophagy activation attenuates renal ischemia-reperfusion injury in rats. Exp Biol Med (Maywood) 240, 1590–1598. 10.1177/1535370215581306 25898836 PMC4935330

[B39] Zhao K. S. (2005). Hemorheologic events in severe shock. Biorheology 42, 463–477. 10.1006/geno.2002.6832 16369084

[B40] Zhao K. S. Huang X. Liu J. Huang Q. Jin C. Jiang Y.. (2002). New approach to treatment of shock–restitution of vasoreactivity. Shock 18, 189–192. 10.1097/00024382-200208000-00017 12166785

[B41] Zhao Z. Si Y. Zhang Y. Du S. Zhang L. Niu C. (2014). Postshock mesenteric lymph drainage ameliorates vascular reactivity and calcium sensitivity through RhoA. J. Surg. Res. 186, 304–309. 10.1016/j.jss.2013.08.017 24075254

[B42] Zhao Z. G. Niu C. Y. Wei Y. L. Zhang Y. P. Si Y. H. Zhang J. (2012). Mesenteric lymph return is an important contributor to vascular hyporeactivity and calcium desensitization after hemorrhagic shock. Shock 38, 186–195. 10.1097/SHK.0b013e31825f1c9b 22683731

