# Supplementary material for: Autophagy Is Involved in Stellate Ganglion Block Reversing Posthemorrhagic Shock Mesenteric Lymph-Mediated Vascular Hyporeactivity
Source: Front Physiol. 2021 Sep 21;12:728191. doi: 10.3389/fphys.2021.728191 (PMC8491623; doi:10.3389/fphys.2021.728191)
Supplement: Supplementary file 1 [file Data_Sheet_1.docx]

**Supplementary Information**

**Autophagy is involved in stellate ganglion block** **reversing post-hemorrhagic shock mesenteric lymph-mediated vascular** **hypo-reactivity**

Chen Wang^1^, Hui-Bo Du^1^, Zhen-Ao Zhao^1, 2, 3^, Jia-Yi Zhai^1^, Li-Min Zhang^1, 2, 3^, Chun-Yu Niu^3, 4 **^, Zi-Gang Zhao^1,2,3*^.

^1^ Institute of Microcirculation, Hebei North University, Zhangjiakou, PR China.

^2^ Pathophysiology Experimental Teaching Center of Basic Medical College, Hebei North University, Zhangjiakou, PR China.

^3^ Key Laboratory of Critical Disease Mechanism and Intervention in Hebei Province, Shijiazhuang and Zhangjiakou, PR China

^4^ Basic Medical College, Hebei Medical University, Shijiazhuang, PR China.

**Correspondence:**

* Zi-Gang Zhao, Institute of Microcirculation, Hebei North University, Diamond South Road 11, Zhangjiakou, Hebei 075000, People’s Republic of China. Tel: +86-18903132966, +86-313-4029223; E-mail: zzghyl@126.com.

** Chun-Yu Niu, Basic Medical College, Hebei Medical University, Zhongshan East Road 361, Shijiazhuang, Hebei 075000, People’s Republic of China. Tel: +86-18931318886, +86-311-86266215; E-mail: [ncylxf@126.com](mailto:ncylxf@126.com).

**Supplementary Figure**


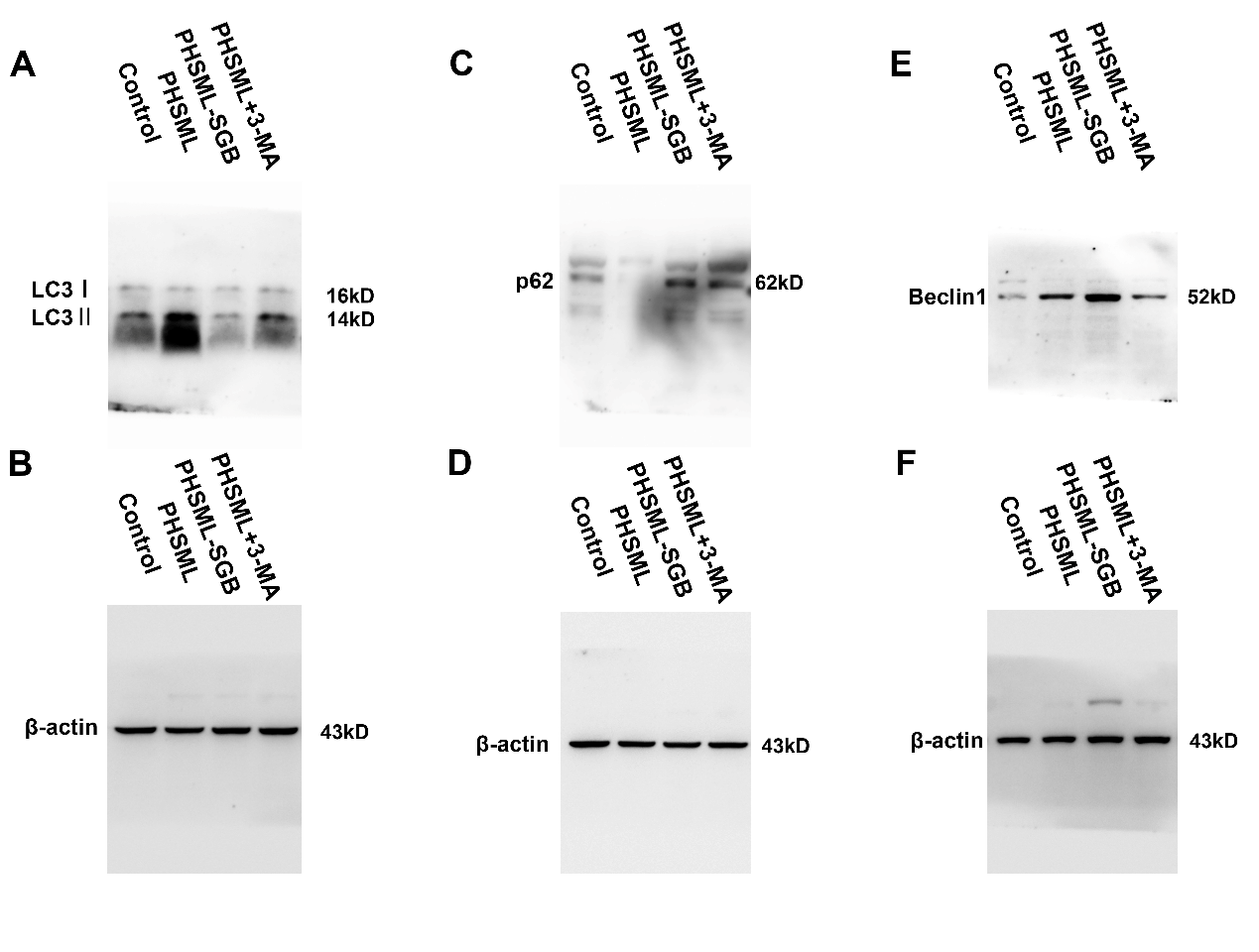


**Supplementary Fig. S1** Original blots related to Fig. 4.
